# The Development Path of the Lighting Industry in Mainland China: Execution of Energy Conservation and Management on Mercury Emission

**DOI:** 10.3390/ijerph15122883

**Published:** 2018-12-15

**Authors:** Zhongguo Li, Puqi Jia, Fu Zhao, Yikun Kang

**Affiliations:** 1 College of Earth and Environmental Sciences, Lanzhou University, Lanzhou 730000, China; jpq@lzu.edu.cn; 2 School of Mechanical Engineering, Purdue University, West Lafayette, IN 47907, USA; fzhao@purdue.edu; 3 Division of Environmental and Ecological Engineering, Purdue University, West Lafayette, IN 47907, USA; 4 Engineering Laboratory for Municipal Waste Pollution Control Technology and Equipment Research, Lanzhou 730000, China; qq332929861@163.com

**Keywords:** incandescent light bulbs, mercury emission, spent fluorescent lamps, energy conservation, light emitting diode, mainland China

## Abstract

The development path of the lighting industry in mainland China was studied in this work. Lighting electricity accounts for about 12% of social electricity consumption in mainland China, while only approximately 15% of electricity is conversed into light when incandescent light bulbs are used. To reduce electrical energy consumption and mercury emission from coal burning in the lighting industry, China worked out a roadmap to replace incandescent light bulbs with energy-saving fluorescent lamps (FLs). However, FL products utilize mercury to give out light and release mercury in their production, consumption and disposal processes. Therefore, the challenges of the lighting industry that mainland China are facing are controlling mercury pollution through the environmentally-friendly producing of fluorescent lamps, effective collecting and treating of spent fluorescent lamps. It was proposed that to effectively reduce energy consumption and mercury pollution, a good way to do this is developing energy-saving and mercury-free light emitting diode lighting industry. The mainland China Government’s strategies to develop lighting industry are worthy of consideration and emulation by other countries.

## 1. Introduction

Around 40% of the word’s electricity is produced from coal [1]. Further, about 66%–83% of electricity and heat have come from coal burning in mainland China [2]. A report on major geographical shift in the global coal market towards Asia said that about half and almost three-quarters of coal demands were in Asia in 2000 and by 2015, respectively [3]. A considerable amount of coal is used to produce electrical power and thermal energy within mainland China. And its burning brings the largest aggregate source of toxic mercury (Hg). When 1 t coal is burned to obtain about 3000 kW·h of electricity, it will generally release 10–1000 mg mercury [4]. On the basis of the China Statistics Yearbook [5], thousands of millions of tons coal are burned in mainland China every year. Consequently, this brings about at least dozens of tons of mercury, which plays a leading role in the emissions of atmospheric mercury in China [6]. Mercury vapor is easily absorbed by the skin, respiratory tract and digestive tract, can accumulate in organisms through food chains and further destroy the central nervous, cardiovascular and immune system, kidney and liver, and even leads to human and other living creature death [7]. Mercury emission is uncontrollable in China because of the coal with the characteristics of low chlorine and high ash [8]. As the biggest energy consumer in the world, China contributes 75% of mercury emissions in East Asia and Southeast Asia, accounting for about 33% of that in the world in total. It makes China the world’s largest atmospheric mercury emitter [9].

As one of the most populous countries, China is one of the world’s largest lighting electricity consumers. Lighting has a non-negligible share (about 12%) of China’s total electricity consumption [10,11]. Incandescent lamps were widely used in the past decades, and their photoelectric conversion efficiency is only about 15%, which makes them an extreme waste of energy. Meanwhile, “If fossil fuels are kept burning at the current rate in the world, they will cross a threshold into environmental ruin by 2036” [12]. Therefore, to decrease electric-related energy consumption and greenhouse gas emission, the State Economic and Trade Commission initiated the China Green Lights Program (CGLP) in 1993, which was officially listed as a vital national energy conservation project during the 9th Five-year Plan period (1996–2000) [10] and was supported by both the United Nations Development Program and the Global Environment Facility.

White light emitting diode (LED) was first successfully developed by Nichia Corporation, Japan in 1996 and gradually expanded after 2000. So, before then, fluorescent lamps (FLs), and compact fluorescent lamps (CFLs) in particular, were used in the energy conservation project in China. Although FLs have a higher photoelectric conversion efficiency than incandescent lamps and reduce mercury emission from electricity consumption, unfortunately FLs rely on mercury as the source of ultraviolet radiation to produce visible light. This presents a new source of mercury emissions. The FLs’ glass tubes are sealed within a small amount of mercury. They do not pose any risk when remaining intact. However, spent fluorescent lamps (SFLs) will eventually be broken and will release the embedded mercury into solid waste stack and, lastly, into the environment [13] if not handled properly. Once broken, CFLs continuously discharged mercury vapor for more than 10 weeks and the level of mercury in a normal room with poor ventilation would exceed the upper exposure safety limit of a healthy adult [13]. It is said that 20.45 tons of mercury were consumed for domestic FL products in 2011 and that most of them have released into the atmosphere [14].

To keep the environment and humans away from the harm caused by mercury and its compounds, the Minamata Convention on Mercury, a global treaty, was signed. It was approved at the fifth session of the Intergovernmental Negotiating Committee (INC) on mercury which was held in Geneva, Switzerland on 19 January, 2013 and was signed afterwards at a Diplomatic Conference (Conference of Plenipotentiaries) in Kumamoto, Japan on 10 October, 2013. Since then, 128 countries and 35 countries have signed and ratified the convention, respectively. China signed the convention in 2013 and became one of the ratification countries in 2016 [15]. The Minamata Convention aims at phasing out extant mercury mines, phasing out and phasing down the use of mercury in lots of products and processes, controlling the emissions of mercury to the air, soil and water, and regulating the dosage of mercury in mining activities. In addition, the convention covers the temporary storage of mercury, the disposal of mercury aforementioned once as waste, the management of mercury-contaminated sites and health issues.

Mercury was released in the production and consumption of FLs, and the disposal process of SFLs, so the Minamata Convention requires a phase out of the use of mercury in certain types of FLs. Under the Minamata Convention on Mercury, mainland China, the world’s largest producer and user of FLs, is facing the serious problem of FLs management. Therefore, the objective of this paper is to find an effective solution for spent FLs management in mainland China and to evaluate the effect of green LED industry on mercury emission and energy consumption.

## 2. Methods

This research was performed by literature collection and data that was obtained from three sources: (1) Government websites, such as The State Council, National Development and Reform Commission, State Environmental Protection Administration of China and Natural Resources Defense Council, Ministry of Industry and Information Technology, Ministry of Science and Technology, Ministry of Ecology and Environment of the People’s Republic of China, United States Environmental Protection Agency, United Nations Environment Programme and International Energy Agency; (2) Government statistics data, e.g., National Bureau of Statistics and GRID-Arendal; (3) Other sources including report and survey, for example, China Industry Information Network. For these data that were used in this work, the units of some data were converted from Chinese custom to international custom, such as, for money, the exchange rate is seven CN yuan for one US dollar. The figures in this work were drawn with Microsoft Visio 2003 (Microsoft Corporation, Redmond, Washington, USA) and/or Microsoft Excel 2003 (Microsoft Corporation, Redmond, Washington, USA).

## 3. Results and Discussion

### 3.1. Energy Conservation and Mercury Emission from FLs

An announcement listed four implementation results of China Green Lights Program which was issued by the National Development and Reform Commission (NDRC) in 2006 [16]. It can be simplified as follows: (1) The continuously growing market share of high-efficiency lighting products reduced 17 million tons of carbon dioxide (carbon) and 530 thousand tons of sulfur dioxide emissions; (2) The product structure of the lighting industry tends to optimize after the promotion for the continuous expansion of the lighting industry; (3) The level of technology and equipment in the lighting industry has been gradually improved, and electric light source production is transforming to automatic operation; (4) The various energy-saving ways demonstrated have laid the foundation for further implementation of the green lighting project.

**Figure 1:**
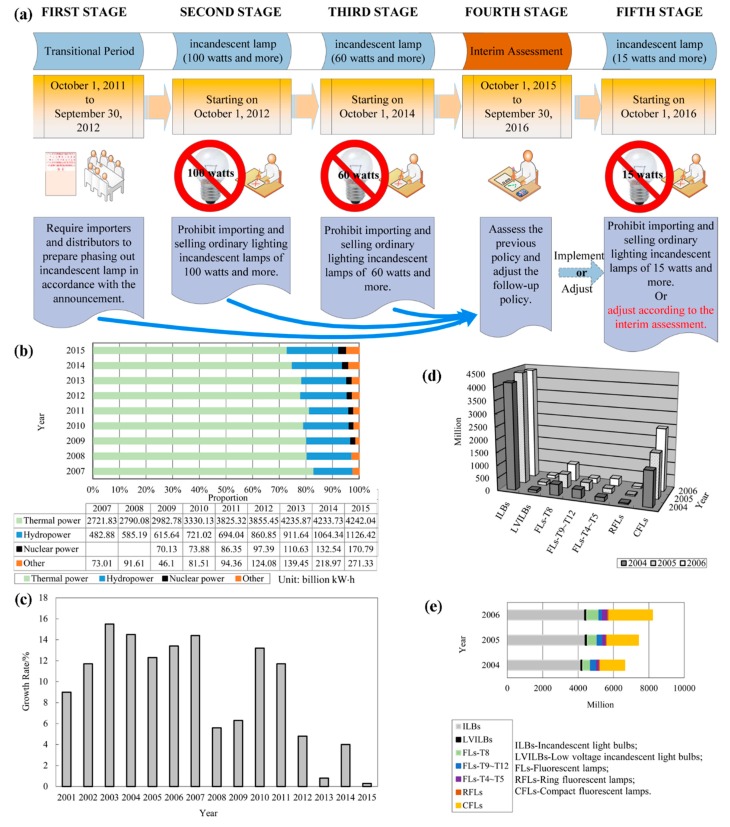
(**a**) Roadmap to phase out incandescent light bulbs in mainland China; (**b**) the reduced share of thermal power as electric power source in China from 2007 to 2015 (Data from [24]); (**c**) the reduced growth rates of electric power output in China at the end of the period from 2001 to 2015 (Data from [2]); (**d**) increased outputs of fluorescent lamps and (**e**) decreased output shares of incandescent light bulbs from 2004 to 2006 (Data from [25,26,27]).

In fact, to improve energy efficiency and to protect the environment, China worked out the Roadmap to phase out incandescent light bulbs (RPILBs) (shown in Figure 1a) in 2011 [17]. As a result, the government of China encouraged manufacturers producing energy-saving lamps to increase the output of FLs rapidly. In 2011, the industry sales of FL products nearly reached $50.5 billion, an increase of 16.6% from 2010. There are more than 10 thousand lighting appliance manufacturers who mainly distribute in southeastern China, e.g., Guangdong, Fujian, Zhejiang and Jiangsu province [18]. It was estimated that the implementation of RPILBs can save about 48 billion kW·h energy, which is equivalent to reducing 48 million tons of carbon dioxide emissions [19].

The year 2012 is the most noteworthy year. Figure 1b shows that since 2012, the share of thermal power has fallen below 78% of electric power because from then on, the implementation of RPILBs worked in China. Further, the share of thermal power came down obviously year by year since 2013. In addition, the growth rate of electric power output shown in Figure 1c declined sharply from 2012 due to the promotion of FLs. From Figure 1b,c, it indicates that RPILBs has worked on energy conservation from 2012. In addition, China has taken great steps, including reducing the share of thermal power as shown in Figure 1b, to respond to global climate change [20].

In fact, the development of FLs in China was very rapid before RPILBs worked out. The products of FLs (e.g., straight FLs, ring FLs, compact FLs, electrodeless FLs) were widely applied to government organizations, schools, buildings, train carriages, families and other indoor and outdoor lightings. The representative outputs and shares of fluorescent lamps and incandescent light bulbs were studied based on the data from 2004 to 2006, in which there were no complicated and manifold influences that were caused by LED because LED was firstly developed in indoor lighting in 2006 [21]. Figure 1d shows that the total output of straight and annular fluorescent lamps was about 1180 million in 2006, and the demand for compact fluorescent lamps (CFLs) has been increasing year by year. Figure 1e shows that the market dominance of incandescent light bulbs was weakened step by step.

**Figure 2:**
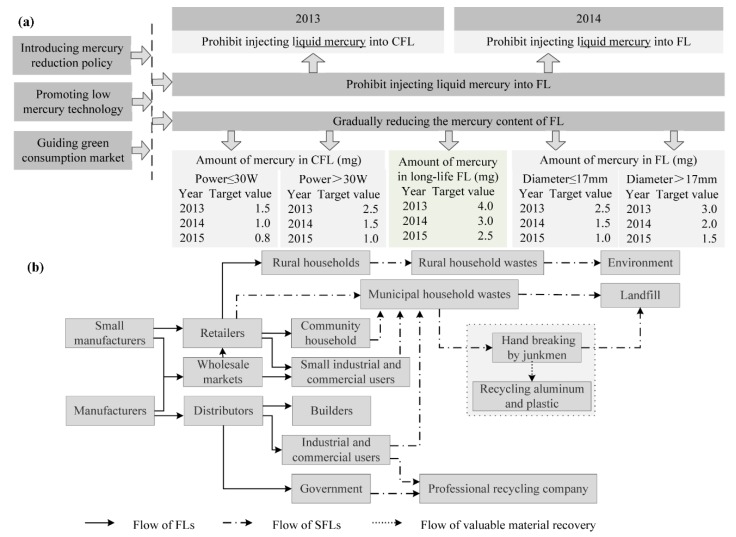
(**a**) Roadmap to Gradually Reduce the Mercury Content in Fluorescent Lamps [29,30] and (**b**) flow chart of production, use of fluorescent lamps and treatment of spent fluorescent lamps in mainland China.

Although the emissions of greenhouse gas and mercury from coal combustion can be reduced when using FLs, the promotion of FLs has also brought the mercury pollution problem [22]. The amount of mercury that is consumed by FL products is large. It was said that 58.6 tons of mercury were consumed in 28.8 billion fluorescent lamps that were produced in 2005 [23]. Further, mercury contents in some special lamps, for example high-intensity discharge (HID) lamps, may be several times those in typical FLs. They should not be ignored when the topic of mercury pollution in FLs is discussed. The Chinese Government has paid attention to this problem and promulgated the “Roadmap to Gradually Reduce the Mercury Content in Fluorescent Lamps to Control Mercury Pollution” (Figure 2a). Additionally, the emissions of mercury from FLs and SFLs have been increasing year by year. Until now, tens of tons of mercury were consumed in hundreds of billions of fluorescent lamp products, according to Figure 1d,e and Figure 2a. In sum, the challenges of producing FLs by an environmentally friendly method and effectively treating SFLs are obviously urgent in China.

### 3.2. Reducing Energy Consumption and Mercury Injected in FLs

The objective of mercury pollution control is clear in China. After the RPILBs was implemented, thermal power, involving greenhouse gas emission and mercury pollution, was effectively limited in mainland China. “Renewable energy development in the 13th Five Year Plan” announced that the proportion of non-fossil energy would take up 15% of primary energy consumption by 2020 and 20% by 2030. The renewable energy consumption target is 730 million tons of standard coal equivalent, of which 580 million tons is commercial volume and 150 million tons is non-commercial by 2020 [28]. This amount is far more than the energy consumption in lighting use. This plan corresponds to the United Nations Framework Convention on Climate Change and responds to the Minamata Convention on Mercury.

FLs belong to mercury vapor lamps and require mercury being injected into the light tube. Although the amount of mercury in FLs can be greatly reduced by an advanced technique, mercury pollution cannot be completely eliminated. Additionally, there are many mercury mine plants in China that produce serious mercury pollution around them [31,32]. The roadmap to reduce the mercury content in FLs has suppressed the mercury production industry in a way and will bring a chance for the government to limit mercury mining in mainland China in the future. At the same time, the use of less mercury in FLs can help limit the development of the mercury consumption market because mercury consumption in FLs occupies a noteworthy share of global mercury consumption [33]. Also, our government is considering establishing a recycling system of SFLs.

### 3.3. Disposal of SFLs

It is said that the output of FLs, the number of SFLs and the disposal rate of SFLs are nearly 7 billion, 100 million and less than 10% annually in mainland China in 2011, respectively [34]. That is because the disposal method of SFLs is not feasible. Most SFLs are sent to landfill and only few SFLs are recycled. Even a fair proportion of SFLs is discarded into the environment. It was reported that the total mercury content in FLs was 20.45 tons in 2011, of which 3.89 tons were incinerated, 15.54 tons were dumped in landfill at the end of life and only 1.02 tons were recycled in mainland China [35]. That is to say that most mercury is eventually released to the atmosphere. Although the Chinese Government has recognized the importance of revising SFLs-related items in the National Hazardous Waste List [36], the social ability to deal with SFLs is seriously deficient.

The current disposal practice for SFLs in China is to treat them as municipal solid wastes (MSW). This was permitted in the sixth item in the “Directory of National Hazardous Wastes (DNHW), 2008 Edition” of China. In mainland China, most SFLs were sent to landfill as household waste, while most of the others were burned together with MSW and only a few of them were recycled, which resulted in about 11% of mercury being released into the air or water [15]. However, according to related standards and requirements, not every landfill considered the future SFLs disposal use when they were built. The collection and treatment systems of leachate were not put in some landfills [37] and many landfills have no gas collection systems [38]. The SFLs that are dumped in landfills will continually release mercury into the surrounding water, soil and even air via the leakage of leachate and the emission of landfill gas [39,40,41]. It was reported that various pollutants including 96 groundwater pollutants, three organic indicators, two visual pollutants and six aggregative pollutants were found in the groundwater around landfills, among which 22 pollutants were considered to be dominant [42].

As an alternative, incineration also has nonnegligible mercury emission issues. It was reported that over 90% of mercury in SFLs may be directly released into the air through incineration by a lack of controls to mercury emission [43]. With the fast market growth of FLs, the emission of mercury from SFLs becomes a serious problem and the effective control of mercury pollution has received increasing researchers’ attentions in China [44]. The quantity of mercury being released into the air can be notably cut down by dry/semi-dry scrub, activated carbon injection and fabric bag filter systems [45,46]. Since then, more discussions about mercury risk in FLs have been reported. Some researchers proposed that the contents of mercury in FLs should be lowered by the manufacturers to effectively cut the cost, avoid serious environmental pollution and easily recycle mercury later [47]. Also, it is necessary to implicate extended producers’ responsibilities on FLs during the management and recycling of FLs [48]. Other researchers paid attention to the comprehensive influence of the policy makers and product manufacturers. In addition, the construction of MSW management facilities and active participation of the public are important for reducing mercury pollution and enhancing the recycling of mercury-containing products [29].

There are only two effective ways to handle SFLs: one is sending SFLs to hazardous waste landfill, and the other is recycling SFLs through a specialized company. However, there are few SFL recycling companies in mainland China. In fact, sending SFLs to hazardous waste landfills becomes the only way for SFL disposal [47]. The detailed flow chart of FLs and SFLs in mainland China is shown in Figure 2b. Last but not least, the management on mercury pollution control in the treatment of SFLs is the challenge that the Chinese Government has to face.

### 3.4. Promoting the Development of the Energy-Saving and Mercury-Free LED Lamp Industry

When the government of China encourages FLs to popularize as energy-saving products, light emitting diode (LED), a new kind of energy-saving two-lead semiconductor light, breaks into the market together with CFLs. As a p–n junction diode, LED emits light in the form of photons when a proper voltage is applied. As a kind of efficient energy-saving lamp, LED is suitable for indoor lighting, street lighting, plant lighting and other lighting [49,50,51]. Further, it can be used in electronic and electrical products as backlight [52]. In fact, the luminescence of one 3 W LED is equivalent to that of one 15 W CFL or one 60 W incandescent light bulb (ILB). Assuming that 4 billion lamps are charged under the power price of $0.07 per kW·h and are lit for 8 h each day in mainland China, the total cost of lighting comes to about $2.5 billion for LEDs, $12.5 billion for CFLs and $50 billion for ILBs every year, respectively. Meanwhile, lighting discharges over 0.12 t mercury (from coal-fired power) for LEDs, 1.38 t mercury (0.58 t from coal-fired power and 0.8 t from spent CFLs—one-quarter of CFLs) for CFLs on the basis of 0.8 mg mercury injected in one CFL and 2.34 t mercury (from coal-fired power) for ILBs every year, respectively. If replacing CFLs and ILBs with LEDs, it makes the monetary and environmental significance obvious.

In fact, like other lamps, LED lamps contain metals [53,54], even some heavy metals (e.g., nickel, lead) [55]. Heavy metal pollution still occurs when LEDs are discarded. However, the pollution of LEDs is much less than that of FLs during collection and disposal. This is because LEDs are not breakable like FLs and involatile heavy metal in a metallic state in LED is more stable than mercury and will not evaporate into the atmosphere under the condition of ambient temperature. Further, the pollution of LED is also less than that of FLs in the work time because LED works under low voltage with less thermal radiation.

Considering the energy efficiency and environmental compatibility, LEDs should take the place of FLs. As the Minamata Convention on Mercury went into effect, LED ushered in greater space for development in the past several years and undoubtedly will become the mainstream lighting in the future. However, the price of an LED lamp is much higher than that of a FL with the same luminous flux. When purchasing lamps, users always notice the power consumption and costs of lamps. They will select a kind of economical lamp after comprehensive analysis of all kinds of lamps. To speed up the implementation process of energy conservation and emission abatement, the Ministry of Science and Technology of China led the establishment of a cross-sectoral, cross-regional and cross-industry “Coordination Leading Group for National Semiconductor Lighting Engineering” on 17 June 2003. From then until the end of 2005, it was the emergency start-up period of the semiconductor lighting project. Starting from the 11th Five-year Plan in 2006, the state promoted the semiconductor lighting project as a major project [56].

In 2007, China’s Ministry of Finance (MOF) associated with National Development and Reform Commission (NDRC) to release “Interim Measures on Administration of Financial Subsidies for Promoting High-Efficiency Lighting Products”. The measures implemented financial subsidies to develop CFLs, LEDs and other highly efficient energy-saving lighting products. According to corresponding regulations, the semiconductor lighting (LED) has been emphatically put forward as an energy product. The related municipal government agencies should purchase LED lamps for residential, building and other applications. LED manufacturers can obtain up to a 20% financial subsidy from the government procurement when the single purchase value is more than $14.5 thousand. Then, local governments implemented LED subsidies actively. For example, Foshan, Dongwan, Zhongshan, Jiangmen and many other cities in Guangdong Province started subsidy projects to help the applications of LED products in 2013. Meanwhile, “Shanghai’s 12th Five-year Plan for semiconductor lighting and industry development” proposed upgrading 20% of Shanghai’s 40 thousand streetlights with LEDs. In addition, Yangzhou city, Jiangsu Province, procured LED lighting products as long ago as 2010.

Industry transition is one important work that the Chinese Government has to carry out in the coming years. For example, Jiangmen city, Guangdong Province, distributed a policy to promote the development of strategic emerging industries containing green lights for subsidizing new expansion or equipment investments in epitaxial wafers, chips, package, applications, substrates, materials and other vital industries involved in the LED industry. Although the LED industry is still in its infancy in China, the lighting industry is owning a good opportunity for industry transition. On 19 May 2015, the State Council released the document of “Made in China 2025” [57], of which its important principles are achieving green development and optimal quality, aiming at comprehensively upgrading Chinese industry to make it more efficient and integrated and building a manufacturing powerful nation with global leadership and influence. After that, the State Council released the “13th Five-year Plan for National Strategic Emerging Industries” on 19 December 2016. The important initiative contents of the plan are promoting green environmental protection, optimizing industrial structure, developing industrial cluster and improving industrial competitiveness.

### 3.5. Enlightenment of the Development Path of the Lighting Industry in Mainland China

The government of China developed incandescent light bulbs before FLs were applied in lighting. Then, the Chinese Government worked out the “Roadmap to Gradually Phase Out Incandescent Light Bulbs” to save energy in November 2011 [17], promulgated the “Roadmap to Gradually Reduce the Mercury Content in Fluorescent Lamps in Mainland China” to reduce mercury emission in February 2013 [58] and signed the Minamata Convention on Mercury that was ratified by the United Nations Environment Programme (UNEP) to protect the environment and human health in October 2013 [59]. China’s action to reduce mercury emission was in advance of that of UNEP, which showed the proactive environmental protection awareness of the Chinese Government. After firstly being developed in indoor lighting in China in 2006, LEDs started to be actively promoted by the Chinese Government. All in all, the Chinese Government is certainly going in the right direction regarding the future lighting industry.

The development path of the lighting industry in China provides great inspiration for the other countries in the world. As the world’s largest consumer for FLs, China faces the most serious mercury pollution. The policies, regulation and methods for the production of FLs, the collection and disposal of SFLs and the measures for the development of LEDs that China is exploring are great references for other countries. It is not necessary for other countries to copy China’s path of lighting development and governance. They can be inspired and then skip some steps that are not suitable for their development, and find the right way to develop the lighting industry based on their national conditions.

## 4. Conclusions

Coal-fired power, contributing to 66%–83% of electricity and heat supplies, brings about at least 6 t of mercury in mainland China every year. The photoelectric conversion efficiency of incandescent light bulbs is only about 15%. To reduce electric consumption and environmental mercury pollution from coal burning during thermal power, energy-saving fluorescent lamps (FLs) were developed in mainland China. However, FLs became a nonnegligible mercury resource because at least 0.8 mg of mercury was injected in every lamp tube during production. So far, there have been hundreds of billions of FLs consumed in mainland China. Although the mercury emission by using FLs is about 60% of that using incandescent light bulbs (ILBs) for lighting, the mercury emission by using LEDs is only about 5% of that using ILBs. So, the policy and management on the production and consumption of FLs and the disposal of SFLs are crucial to reduce mercury emission in mainland China. Meanwhile, developing LEDs is a good way to realize an energy-saving and mercury-free lamp industry. The development and experience of the lighting industry in mainland China is a reference for the future development direction of other countries.
